# Frequent cyclic variation of heart rate is associated with left ventricular diastolic dysfunction in patients without ischemia

**DOI:** 10.1002/hsr2.463

**Published:** 2021-12-21

**Authors:** Takanori Yaegashi, Manabu Nakano, Yoshiharu Murata

**Affiliations:** ^1^ Department of Cardiology Noto General Hospital Nanao Japan

**Keywords:** cyclic variation of heart rate, left ventricular diastolic dysfunction, quantitative gated single‐photon emission computed tomography, sleep apnea syndrome

## Abstract

**Background:**

Cyclic variation of heart rate (CVHR) associated with sleep‐disordered breathing reflects cardiac autonomic responses to apneic/hypoxic stress. However, the association of CVHR with cardiac function is unclear.

**Methods:**

We investigated a total of 181 patients who underwent both 24‐hour Holter electrocardiography (ECG) and quantitative gated single‐photon emission computed tomography (SPECT) myocardial functional imaging, excluding patients with atrial fibrillation, myocardial infarction, structural heart disease, and implantable devices, from January 2017 to July 2018. The number of CVHR per hour (CVHR index) in sleeping‐time Holter ECG was compared with the parameters of left ventricular (LV) systolic and diastolic functions assessed by cardiac SPECT functional imaging, peak filling rate (PFR), first‐third mean filling rate (1/3 MFR), and time to peak filling rate (TTPF).

**Results:**

In all patients, the CVHR index was not associated with any parameters of cardiac functions. However, in a propensity score–matched subgroup of patients without ischemia (N = 39), the CVHR index was negatively correlated with PFR (r = −0.35, *P* < .05) and 1/3 MFR (r = −0.37, *P* < .05) but positively correlated with TTPF (r = 0.43, *P* < .01) and was not correlated with LV ejection fraction. Multivariate linear regression analysis revealed that high CVHR index was independently associated with LV diastolic dysfunction, even after adjusting for the relative wall thickness and LV mass index assessed by echocardiography.

**Conclusion:**

These results indicate that the high frequency of CVHR in sleeping time is associated with LV diastolic dysfunction in nonischemic patients, irrespective of LV geometry.

## 1 INTRODUCTION

Sleep‐related breathing disorders are associated with cardiac diastolic dysfunction. Previous studies demonstrated that apnea‐hypopnea index (AHI) in patients with obstructive sleep apnea (OSA) was correlated with left ventricular (LV) diastolic dysfunction evaluated by echocardiography.^1^, ^2^ However, polysomnography, which is the gold standard for diagnosis, is rather troublesome because the test includes electroencephalography that requires a one‐night stay in a medical facility. Episodes of OSA are accompanied by a characteristic heart rate alteration that consists of bradycardia during apnea, followed by abrupt tachycardia on its cessation, which is known as cyclic variation of heart rate (CVHR).^3^, ^4^, ^5^ CVHR creates dips in the R‐R interval series. Recently, CVHR has been available by analyzing 24‐hour Holter electrocardiography (ECG). CVHR index, which is the CVHR per hour in sleeping‐time Holter ECG, has been closely correlated with AHI.^5^, ^6^, ^7^ The automated detection of CVHR index from Holter ECG provides a powerful screening tool for OSA, even in older patients and in those with dementia and cardiac autonomic dysfunction. However, it is unclear whether CVHR is associated with cardiac function. This study aimed to investigate whether CVHR is associated with LV systolic or diastolic function.

Furthermore, myocardial ischemia itself causes not only LV systolic dysfunction but only LV diastolic dysfunction.^8^, ^9^, ^10^, ^11^, ^12^ Therefore, the comorbidity of myocardial ischemia was expected to mask the relationship between CVHR and LV diastolic dysfunction. To address this issue in this study, we also investigated the subgroup analysis in patients with and without myocardial ischemia.

## 2 METHODS

### 2.1 Study population and ethical considerations

**FIGURE 1:**
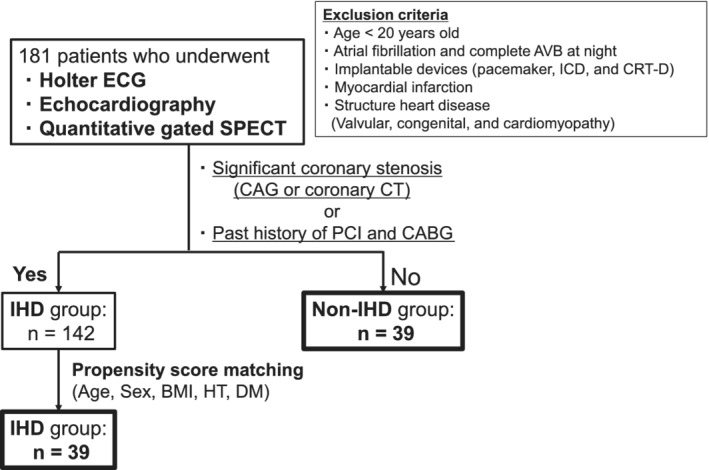
Flowchart of the study. AVB, atrioventricular block; BMI, body mass index; CABG, coronary artery bypass grafting; CAG, coronary angiography; CRT‐D, cardiac resynchronizing therapy with defibrillation; CT, computed tomography; DM, diabetes mellitus; ECG, electrocardiogram; HT, hypertension; ICD, implantable cardioverter defibrillator; IHD, ischemic heart disease; PCI, percutaneous coronary intervention; SPECT, single‐photon emission computed tomography

We retrospectively investigated a total of 181 hospitalized patients who underwent Holter ECG, echocardiography, and quantitative gated single‐photon emission computed tomography (SPECT) from January 2017 to July 2018, after excluding patients who met the following criteria: age <20 years, atrial fibrillation and complete atrioventricular block at night, implantable devices, myocardial infarction, and structural heart disease. Since the LV diastolic function is impaired in an ischemic heart, to examine the impact of ischemia on cardiac function, we divided the 181 patients into the ischemic heart disease (IHD) group and nonischemic heart disease (non‐IHD) group (Figure 1). IHD group included 142 patients with either significant coronary stenosis confirmed by coronary angiography or multidetector computed tomography or history of coronary revascularization, such as percutaneous coronary intervention and coronary artery bypass graft, whereas other patients were assigned to non‐IHD group. This study and the retrospective data used were approved by the Bioethical Committee of Noto General Hospital and conducted in accordance with the ethical standards in the 1964 Declaration of Helsinki and its later amendments.

### 2.2 Assessment of CVHR

ECG signals were obtained via the Holter LS‐300 system at a sampling frequency of 125 Hz. A 24‐hour Holter automatic arrhythmia analysis system (SCM‐850S, Fukuda Denshi) identified all R‐wave positions and excluded abnormal beats, such as ventricular and supraventricular ectopic complexes and artifacts. The algorithm to detect CVHR automatically was prepared as previously reported.^6^ The CVHR index was calculated as the mean number of CVHR per hour in bed, which was obtained from the behavior records of subjects.

### 2.3 Evaluation of echocardiographic findings

All patients underwent transthoracic echocardiography (Sequoia, Siemens Medical Solutions USA Inc., Mountain View, California) by a technician. LV and left atrial internal dimensions and LV wall thickness were measured at end‐diastole and end‐systole in accordance with the recommendations of the American Society of Echocardiography (ASE) and the European Association of Cardiovascular Imaging (EACVI).^13^ Both two‐dimensional mode and M‐mode echocardiography were performed in all patients. LV mass was calculated according to the anatomically validated Cube formula.^13^ LV mass index (LVMI) was calculated by dividing the LV mass by the body surface area. Relative wall thickness (RWT) was calculated using the following formula: RWT = (2 × thickness of LV posterior wall/LV diameter at end diastole). Pulsed Doppler measurements of LV diastolic inflow were obtained under 2‐dimensional echo‐guidance. An LV diastolic filling pattern was recorded from the apical transducer position with the patients in a partial left lateral decubitus position, and the sample volume situated between the mitral leaflet tips. The early diastolic transmural flow velocity (E velocity) and the late (atrial) transmural flow velocity (A velocity) were recorded, and the early to late diastolic transmural flow velocity (E/A) ratio and deceleration time (DcT) were calculated from three consecutive cardiac cycles.

### 2.4 Evaluation of quantitative gated SPECT findings

ECG‐gated single‐photon emission computed tomography (SPECT) was performed at rest and during sinus rhythm using thallium‐201 chloride (111 MBq) or technetium‐99 m tetrofosmin (740 MBq) as a tracer. At 60 minutes after intravenous tracer injection, ECG‐gated SPECT data were obtained for 60 seconds per projection from 30 projections during a 180° rotation using a two‐headed gamma camera with a low‐energy, high‐resolution parallel‐hole collimator. A cardiac cycle was divided into 16 frames. The data were stored in a 64 × 64‐word matrix nuclear computer system, and no attenuation or scatter correction was applied to this protocol. For the data analysis, the quantitative gated SPECT program (Cedars‐Sinai Medical Center, Los Angeles, California) was applied to process short‐axis tomograms to determine the LV end diastolic volume, LV end‐systolic volume, and LV ejection fraction (LVEF). Peak filling rate (PFR), first‐third mean filling rate (1/3 MFR), and time to peak filling rate (TTPF) were also obtained as the diastolic functional parameters.^14^, ^15^

### 2.5 Statistical analysis

Normally distributed continuous variables were presented as mean ± standard deviation (SD) for each group unless stated and analyzed by unpaired *t*‐test for two‐group comparisons. Categorical variables were compared using the chi‐square test. Pearson's correlation analysis was used to evaluate the correlations between the parameters. The multivariable linear regression analysis was employed to identify the independent parameter for impaired LV diastolic function. A propensity score was used to match the subgroups in a 1:1 ratio and calculated using logistic regression. Covariates with a *P*‐value <.20, as determined by univariate analysis, were used to generate the propensity score, which included age, body mass index (BMI), hypertension, and diabetes. In addition, sex was included in the matching variable, considering the possibility of imbalance after propensity score matching. A *P*‐value <.05 was considered to indicate statistical significance. Statistical analyses were performed using GraphPad Prism 6 statistical software (GraphPad Software, San Diego, California) and EZR (Saitama Medical Center, Jichi Medical University, Saitama, Japan), which is a graphical user interface for the R software program (The R foundation for Statistical Computing, Vienna, Austria).^16^

## 3 RESULTS

**FIGURE 2:**
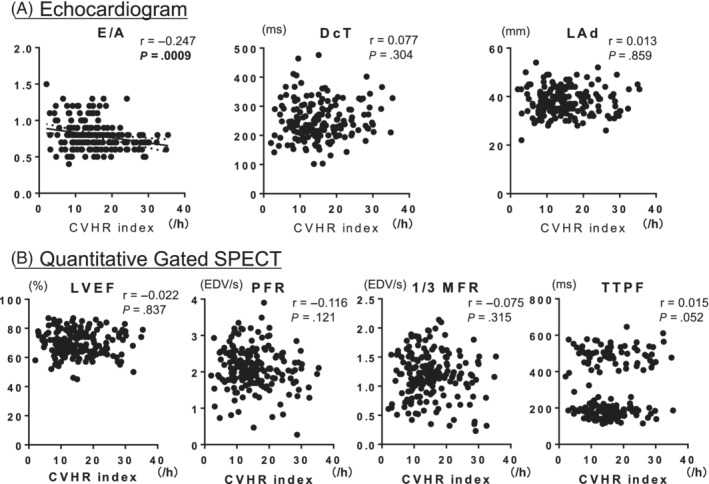
Relationship between CVHR index and LV systolic and diastolic function in all subjects. CVHR, cyclic variation of heart rate; DcT, declaration time; E/A, early to late (atrial) diastolic transmural flow velocity; LVEF, left ventricular ejection fraction; 1/3 MFR, first‐third mean filling rate; PFR, peak filling rate; r, correlation coefficient; TTPF, time to peak filling rate

The flowchart of this study is shown in Figure 1. The clinical characteristics of the 181 patients of this study are presented in Table 1 and Table S1. 38% of patients had diabetes, and 88% had hypertension. The Holter ECG showed that the mean CVHR index was 15 per hour. The average values of LVMI and RWT were within the normal range. In these subjects, the CVHR index was associated with E/A ratio but not associated with DcT, left atrial diameter (LAD), LVEF, PFR, 1/3 MFR, or TTPF (Figure 2).

**TABLE 1:** Clinical characteristics of total and a propensity score–matched comparable subgroups

		Total (n = 181)	Propensity score–matched subgroup		*P*‐value
			Non‐IHD (n = 39)	IHD (n = 39)	
Age (years)		72.7 ± 10.0	70.6 ± 10.8	71.2 ± 11.7	*P* = .83
Male, n (%)		126 (69.6%)	22 (56.4%)	26 (66.7%)	*P* = .36
BMI (kg/m^2^)		24.1 ± 3.3	23.4 ± 3.9	23.7 ± 3.2	*P* = .66
DM, n (%)		68 (37.6%)	4 (10.3%)	2 (5.1%)	*P* = .40
HT, n (%)		160 (88.3%)	30 (76.9%)	30 (76.9%)	*P* > .99
Holter ECG					
Mean HR (bpm)		67.5 ± 8.6	70.3 ± 8.0	68.6 ± 8.9	*P* = .37
CVHR index (/h)		15.3 ± 7.1	15.6 ± 6.7	16.5 ± 7.7	*P* = .61
Echocardiogram					
LV wall thickness	LVMI (g/m^2^)	82.5 ± 23.8	87.1 ± 20.5	84.0 ± 24.8	*P* = .55
	RWT	0.39 ± 0.07	0.40 ± 0.08	0.39 ± 0.07	*P* = .55
LV diastolic function	E/A ratio	0.80 ± 0.20	0.88 ± 0.22	0.79 ± 0.19	*P* = .06
	DcT(ms)	249.2 ± 64.5	228.3 ± 49.4	253.6 ± 69.0	*P* = .07
LAD (mm)		38.1 ± 5.5	37.0 ± 5.9	38.7 ± 5.8	*P* = .19
QGS					
LV systolic function	LVEF (%)	70.0 ± 8.5	70.6 ± 8.3	70.6 ± 8.9	*P* = .99
LV diastolic function	PFR (EDV/s)	2.11 ± 0.57	2.14 ± 0.69	2.05 ± 0.50	*P* = .53
	1/3 MFR (EDV/s)	1.19 ± 0.39	1.25 ± 0.43	1.18 ± 0.38	*P* = .44
	TTPF (ms)	292 ± 159	254 ± 144	316 ± 162	*P* = .08

*Note*: Values are given as mean ± SD or absolute and relative (in percent) frequencies, respectively. *P*‐value is the significance level of the unpaired *t*‐test to compare the values of Non‐IHD group and IHD group.

Abbreviations: 1/3 MFR, first‐third mean filling rate; BMI, body mass index; CVHR, cyclic variation of heart rate; DcT, deceleration time; DM, diabetes mellitus; E/A, early to late (atrial) diastolic transmural flow velocity; HR, heart rate; HT, hypertension; IHD, ischemic heart disease; LAD, left atrial diameter; LVEF, left ventricular ejection fraction; LVMI, left ventricular mass index; PFR, peak filling rate; QGS, quantitative gated single‐photon emission computed tomography; RWT, relative wall thickness; SD, standard deviation; TTPF, time to peak filling rate.

**FIGURE 3:**
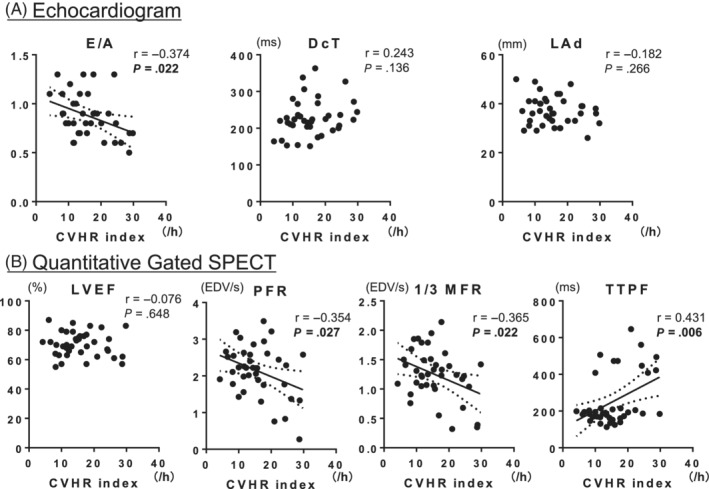
Relationship between CVHR index and LV systolic and diastolic function in the subgroup of nonischemic patients. CVHR, cyclic variation of heart rate; DcT, declaration time; E/A, early to late (atrial) diastolic transmural flow velocity; IHD, ischemic heart disease; LAd, left atrial diameter; LVEF, left ventricular ejection fraction; 1/3 MFR, first‐third mean filling rate; PFR, peak filling rate; r, correlation coefficient; TTPF, time to peak filling rate

**FIGURE 4:**
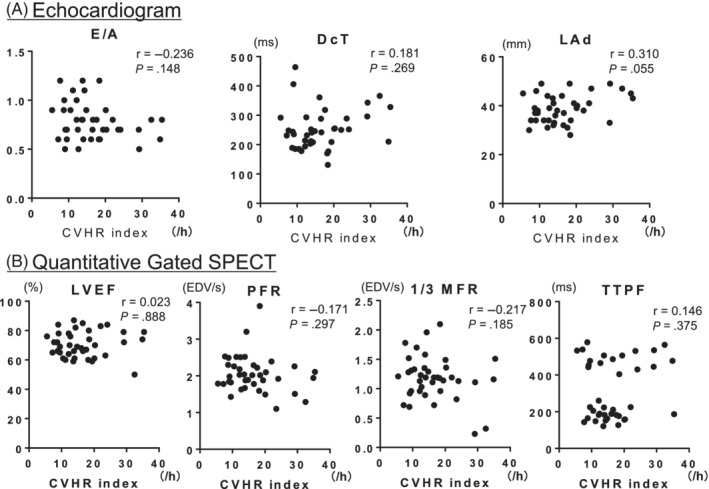
Relationship between CVHR index and LV systolic and diastolic function in the subgroup of ischemic patients. CVHR, cyclic variation of heart rate; DcT, declaration time; E/A, early to late (atrial) diastolic transmural flow velocity; IHD, ischemic heart disease; LAd, left atrial diameter; LVEF, left ventricular ejection fraction; 1/3 MFR, first‐third mean filling rate; PFR, peak filling rate; r, correlation coefficient; TTPF, time to peak filling rate

There were statistical differences in some parameters of clinical backgrounds between the non‐IHD group and IHD groups (Table S1). To avoid selection bias, we used propensity score matching to select comparable groups of age, sex, BMI, hypertension, and diabetes. Thus, each of the 39 patients was selected for further analysis. There was no statistical difference in any parameters of clinical backgrounds between the non‐IHD group and IHD groups (Table 1). In the propensity score‐matched subgroup of patients without IHD, the CVHR index was negatively correlated with E/A ratio, PFR, and 1/3 MFR but positively correlated with TTPF and was not associated with DcT, Lad, and LVEF (Figure 3). Meanwhile, in the subgroup of patients with IHD, the CVHR index was not associated with E/A ratio, DcT, LAd, LVEF, PFR, 1/3 MFR, or TTPF (Figure 4).

The univariate analysis (Table 2) demonstrated that, in patients without IHD, any parameters of age, sex, BMI, hypertension, and diabetes were not associated with LV diastolic function. LVMI and RWT were associated with decreased PFR and increased TTPF. In contrast, the CVHR index significantly correlated with all LV diastolic function parameters. The multivariate linear regression analysis (Table 2) revealed that only CVHR index was a significant independent parameter that was associated with decreased PFR and 1/3 MFR or increased TTPF after adjustment for LVMI and RWT.

**TABLE 2:** Univariable and multivariable linear regression analyses for E/A, PFR, 1/3 MFR, and TTPF in patients without IHD

Variables	E/A ratio				PFR				1/3 MFR				TTPF			
	Univariate		Multivariate		Univariate		Multivariate		Univariate		Multivariate		Univariate		Multivariate	
	r	*P*	β	*P*	r	*P*	β	*P*	r	*P*	β	*P*	r	*P*	β	*P*
Age	−0.268	.109			−0.106	.520			−0.097	.559			0.162	.324		
Sex	0.237	.158			−0.058	.728			−0.072	.664			0.112	.497		
BMI	−0.207	.220			−0.065	.693			−0.067	.683			0.033	.843		
HT	−0.142	.402			−0.052	.755			0.033	.844			0.171	.296		
DM	−0.005	.975			−0.038	.817			0.074	.652			−0.078	.636		
LVMI (g/m^2^)	−0.072	.672	0.154	.358	−0.414	**.009**	−0.309	.065	−0.274	.092	−0.175	.316	0.352	**.028**	0.300	.074
RWT	−0.491	**.002**	−0.507	**.005**	−0.433	**.006**	−0.206	.225	−0.356	**.026**	−0.193	.285	0.346	**.031**	0.100	.556
CVHR index (/hour)	−0.375	**.022**	−0.247	0.108	−0.354	**.027**	−0.303	**.043**	−0.365	**.022**	−0.319	**.046**	0.431	**.006**	0.406	**.008**

*Note*: Bold values shown *P*‐value <0.05.

Abbreviations: 1/3 MFR, first‐third mean filling rate; BMI, body mass index; CVHR, cyclic variation of heart rate; DM, diabetes mellitus; E/A, early to late (atrial) diastolic transmural flow velocity; HT, hypertension; IHD, ischemic heart disease; LVMI, left ventricular mass index; PFR, peak filling rate; r, correlation coefficient; RWT, relative wall thickness; TTPF, time to peak filling rate; β, standard coefficient.

## 4 DISCUSSION

The major findings of this retrospective study are as follows: (a) CVHR index was negatively correlated with the parameter indicating LV diastolic function evaluated using gated SPECT imaging in patients without ischemia. (b) Multivariate linear regression analysis revealed that high CVHR index was independently associated with LV diastolic dysfunction, after adjusting for the LVMI and RWT.

Heart failure with preserved ejection fraction (HFpEF) is a clinical syndrome in which patients have symptoms and signs of heart failure with normal or near‐normal LVEF. Now, it accounts for 56% of patients with heart failure, and its prevalence is increasing.^17^ LV diastolic dysfunction is one of the primary pathophysiological characteristics that underlie HFpEF.

OSA has been associated with diastolic dysfunction and increased LVMI, which is the result from episodes of repetitive hypoxia during sleep and increases in afterload.^18^, ^19^ Usui et al demonstrated that severe OSA itself may directly contribute LV diastolic function irrespective of arterial stiffness, LV geometries, or blood pressure.^1^ LV pressure overload, which is caused by activation of sympathetic nerve activation during sleep^20^ and recurrent episodes of negative intrathoracic pressure during apneic episodes,^21^ may also cause alteration in LV relaxation properties. In addition, increased central blood pressure, which plays an important role in the occurrence of HFpEF due to abnormal ventricular‐vascular coupling,^22^ may contribute to the etiology of diastolic dysfunction in patients with OSA. Therefore, early detection of sleep‐related breathing disorders is important to prevent progression toward HFpEF.

The central sleep apnea (CSA) is another class of sleep‐related breathing disorder. Like the OSA, the CSA also tends to be accompanied with HFpEF.^23^ However, previous study reported that in CSA patients with HFpEF, LV diastolic dysfunction was correlated with increasing age but not with peripheral and central hypercapnic ventilatory responses or severity of central sleep apnea.^24^

Definite diagnosis of OSA requires all‐night laboratory polysomnographic examination, which is occasionally inconvenient to perform. Thus, the Holter ECG‐based screening for sleep apnea could be an easy supportive method. CVHR index in sleeping‐time Holter ECG was reported to be closely correlated with AHI in full polysomnography.^5^, ^6^, ^7^

The LV diastolic dysfunction of the local myocardium exposed to ischemia due to organic coronary artery stenosis, now on, in the past, is thought to persist regardless of the presence of OSA.^8^, ^11^, ^12^ Previous studies demonstrating the relationship between AHI and LV diastolic dysfunction were excluded patients with a history of cardiovascular disease.^1^, ^2^ Therefore, the subjects in the previous studies are similar to those in the non‐IHD group of our study. On the other hand, hypertension and hyperglycemia are individually important risk factors for LV hypertrophy.^25^ Another report showed that severe OSA and metabolic syndrome could cause LV diastolic dysfunction individually and synergistically.^26^ No articles have directly been associated with AHI in patients with ischemic heart disease, but myocardial ischemia might be considered an influential factor enough to mask the contribution of CVHR/OSA to LV diastolic function.

ECG‐gated SPECT can quantitatively and objectively assess LV systolic (LVEF) and diastolic (PFR, 1/3 MFR, and TTPF) function. Echocardiography is noninvasive and frequently used for evaluation of LV function; however, the intra‐ and interoperator variation in measurement tend to be larger. In contrast, PFR, 1/3 MFR, and TTPF obtained from gated SPECT have been highly correlated with LV end diastolic pressure (LVEDP), which was measured using the invasive method of cardiac catheterization.^27^ When diastolic dysfunction worsens, LVEDP increases with a negatively correlated reduction in PFR and 1/3 MFR, whereas there is a positive correlation with increasing TTPF. In contrast, the deceleration time of mitral E‐wave and E to A ratio measured using echocardiography as LV diastolic dysfunction do not present a linear correlation with LVEDP. Therefore, our study using SPECT may have more sensitively indicated the relationship between LV diastolic dysfunction and sleep‐related breathing disorder compared to previous studies using echocardiography.

## 5 LIMITATIONS

The present study has several limitations. First, this study collected single hospital‐based samples, and the number of study sample became relatively small. Therefore, the existence of referral bias should be considered. Second, CVHR index was less certain than AHI because the sleeping time was self‐reported and was not judged by electroencephalographic findings. Third, patients with atrial fibrillation or implanted pacing devices are not applicable because of the loss of the physiological significance of the CVHR index. Fourth, it is unclear whether CVHR index is related to heart failure caused by the diastolic dysfunction, as few patients had moderate to severe heart failure in our population. Fifth, the e′ value and left atrial volume index (LAVI) evaluated by echocardiography were missing in many cases, which made it difficult to classify them into the severity of LV diastolic function (grade I‐III) recommended by the ASE/EACVI guideline.^28^ Finally, our study was cross‐sectional, and the causal relationship between CVHR index and LV diastolic function cannot be derived. Further prospective studies are desirable such as the comparison of healthy volunteer with IHD patients and the interventions of PCI or CPAP treatment.

## 6 CONCLUSION

High frequency of CVHR in sleeping time is associated with LV diastolic function in nonischemic patients, irrespective of LV geometry. CVHR determined by Holter ECG may be a useful screening index that reflects early LV diastolic dysfunction in patients without structural and ischemic heart disease.

## CONFLICT OF INTEREST

The authors have no conflict of interest.

## AUTHOR CONTRIBUTION

Conceptualization: Takanori Yaegashi.

Formal analysis: Takanori Yaegashi.

Investigation: Takanori Yaegshi.

Methodology: Takanori Yaegshi.

Supervision: Manabu Nakano and Yoshiharu Murata.

Writing—original draft preparation: Takanori Yaegashi.

Writing—review and editing: Manabu Nakano.

All authors have read and approved the final version of the manuscript.

Dr Takanori Yaegashi had full access to all of the data in this study and takes complete responsibility for the integrity of the data and the accuracy of the data analysis.

## TRANSPARENCY STATEMENT

Takanori Yaegashi affirms that this manuscript is an honest, accurate, and transparent account of the study being reported; that no important aspects of the study have been omitted; and that any discrepancies from the study as planned (and, if relevant, registered) have been explained.

## Supporting information


**Table S1.** Clinical characteristics of total and subgroups.

## Data Availability

The data that support the findings of this study are available from the corresponding author upon reasonable request.
