# Supplementary material for: Frequent cyclic variation of heart rate is associated with left ventricular diastolic dysfunction in patients without ischemia
Source: Health Sci Rep. 2021 Dec 21;4(4):e463. doi: 10.1002/hsr2.463 (PMC8691488; doi:10.1002/hsr2.463)
Supplement: Supplementary file 1 — Table S1. Clinical characteristics of total and subgroups. [file HSR2-4-e463-s001.docx]

**SUPPLEMENTAL MATERIALS**

**Frequent Cyclic Variation of Heart Rate Reflects Left Ventricular Diastolic Dysfunction in Patients Without Ischemia**

Takanori Yaegashi, MD, PhD; Manabu Nakano, MD, Yoshiharu Murata, MD

**Supplemental Table1. Clinical characteristics of total and subgroups**

|  | | | **Total　(n=181)** | **Non-IHD (n=39)** | **IHD**  **(n=142)** | **P-value** |
| --- | --- | --- | --- | --- | --- | --- |
| Age (years) | | | 72.7 ± 10.0 | 70.6 ± 10.8 | 73.3 ± 9.7 | p = 0.13 |
| Male, n (%) | | | 126 (69.6%) | 22 (56.4%) | 104 (73.2%) | p = 0.43 |
| BMI (kg/m^2^) | | | 24.1 ± 3.3 | 23.4 ± 3.9 | 24.3 ± 3.1 | p = 0.11 |
| DM, n (%) | | | 68 (37.6%) | 4 (10.3%) | 64 (45.1%) | ***p < 0.0001*** |
| HT, n (%) | | | 160 (88.3%) | 30 (76.9%) | 130 (91.5%) | ***p = 0.01*** |
| **Holter ECG** | | |  |  |  |  |
| Mean HR (bpm) | | | 67.5 ± 8.6 | 70.3 ± 8.0 | 66.7 ± 8.6 | ***p = 0.02*** |
| CVHR index (/h) | | | 15.3 ± 7.1 | 15.6 ± 6.7 | 15.1 ± 7.2 | p = 0.70 |
| **Echocardiogram** | | |  |  |  |  |
| LV wall thickness | LVMI (g/m^2^) | | 82.5 ± 23.8 | 87.1 ± 20.5 | 81.3 ± 24.6 | p = 0.18 |
|  | RWT | | 0.39 ± 0.07 | 0.40 ± 0.08 | 0.39 ± 0.07 | p = 0.58 |
| LV diastolic function | | E/A ratio | 0.80 ± 0.20 | 0.88 ± 0.22 | 0.77 ± 0.19 | ***p = 0.005*** |
|  |  | DcT(ms) | 249.2 ± 64.5 | 228.3 ± 49.4 | 255.1 ± 67.1 | ***p = 0.02*** |
| LAD (mm) | | | 38.1 ± 5.5 | 37.0 ± 5.9 | 37.0 ± 5.9 | p = 0.16 |
| **QGS** | | |  |  |  |  |
| LV systolic function | LVEF (%) | | 70.0 ± 8.5 | 70.6 ± 8.3 | 69.9 ± 8.5 | p = 0.63 |
| LV diastolic function | PFR  (EDV/s) | | 2.11 ± 0.57 | 2.14 ± 0.69 | 2.10 ± 0.54 | p = 0.73 |
|  | 1/3 MFR  (EDV/s) | | 1.19 ± 0.39 | 1.25 ± 0.43 | 1.18 ± 0.38 | p = 0.30 |
|  | TTPF (ms) | | 292 ± 159 | 254 ± 144 | 302 ± 162 | p = 0.09 |

Note: Values are given as mean ± SD or absolute and relative (in percent) frequencies, respectively. P-value is the significance level of the unpaired t-test to compare the values of non-IHD group and IHD group. Abbreviations: BMI, body mass index; CVHR, cyclic variation of heart rate; DcT, deceleration time; DM, diabetes mellitus; E/A, early to late (atrial) diastolic transmural flow velocity; HR, heart rate; HT, hypertension; IHD, ischemic heart disease; LAD, left atrial diameter; LVEF, left ventricular ejection fraction; LVMI, left ventricular mass index; 1/3 MFR, first-third mean filling rate; PFR, peak filling rate; QGS, quantitative gated single-photon emission computed tomography; RWT, relative wall thickness; SD, standard deviation; TTPF, time to peak filling rate.
